# Intraclonal mating occurs during tsetse transmission of *Trypanosoma brucei*

**DOI:** 10.1186/1756-3305-2-43

**Published:** 2009-09-21

**Authors:** Lori Peacock, Vanessa Ferris, Mick Bailey, Wendy Gibson

**Affiliations:** 1 School of Biological Sciences University of Bristol, Bristol BS8 1UG, UK; 2 Department of Clinical Veterinary Science, University of Bristol, Langford, Bristol BS40 7DU, UK

## Abstract

**Background:**

Mating in *Trypanosoma brucei *is a non-obligatory event, triggered by the co-occurrence of different strains in the salivary glands of the vector. Recombinants that result from intra- rather than interclonal mating have been detected, but only in crosses of two different trypanosome strains. This has led to the hypothesis that when trypanosomes recognize a different strain, they release a diffusible factor or pheromone that triggers mating in any cell in the vicinity whether it is of the same or a different strain. This idea assumes that the trypanosome can recognize self and non-self, although there is as yet no evidence for the existence of mating types in *T. brucei*.

**Results:**

We investigated intraclonal mating in *T. b. brucei *by crossing red and green fluorescent lines of a single strain, so that recombinant progeny can be detected in the fly by yellow fluorescence. For strain 1738, seven flies had both red and green trypanosomes in the salivary glands and, in three, yellow trypanosomes were also observed, although they could not be recovered for subsequent analysis. Nonetheless, both red and non-fluorescent clones from these flies had recombinant genotypes as judged by microsatellite and karyotype analyses, and some also had raised DNA contents, suggesting recombination or genome duplication. Strain J10 produced similar results indicative of intraclonal mating. In contrast, trypanosome clones recovered from other flies showed that genotypes can be transmitted with fidelity. When a yellow hybrid clone expressing both red and green fluorescent protein genes was transmitted, the salivary glands contained a mixture of fluorescent-coloured trypanosomes, but only yellow and red clones were recovered. While loss of the *GFP *gene in the red clones could have resulted from gene conversion, some of these clones showed loss of heterozygosity and raised DNA contents as in the other single strain transmissions. Our observations suggest that many recombinants are non-viable after intraclonal mating.

**Conclusion:**

We have demonstrated intraclonal mating during fly transmission of *T. b. brucei*, contrary to previous findings that recombination occurs only when another strain is present. It is thus no longer possible to assume that *T. b. brucei *remains genetically unaltered after fly transmission.

## Background

Genetic exchange and the production of hybrid trypanosomes can occur when two different strains of *Trypanosoma brucei *are co-transmitted through the tsetse fly vector [1,2]. In crosses to date, all subspecies of *T. brucei *have proved compatible, except *T. b. gambiense *group 1, excluded by its poor transmissibility in the commonly-used laboratory tsetse fly, *Glossina morsitans morsitans *[3]. Thus there would appear to be no subspecific barriers to mating, but nevertheless some kind of mating type restriction is thought to exist, because intraclonal mating occurs rarely and has been detected only in the presence of mating trypanosomes of different strains [4,5]. The hypothesis put forward is that some kind of diffusible factor or pheromone is produced by trypanosomes on recognition of non-self, which then triggers all trypanosomes in the vicinity to mate. Clearly, for this to work, trypanosomes must be able to recognize self and non-self, but mating types have not yet been described in *T. brucei*. A simple two-sex mating system was ruled out by the three-way cross carried out by Turner and colleagues [6], suggesting that the mating system of this diploid organism probably involves multiple mating types.

Previous studies on intraclonal mating have relied on genotyping individual clones after fly transmission, but the laborious and time-consuming nature of this work has limited the number of individual clones analysed, perhaps contributing to the failure to detect recombinants. For example, Tait et al [5] examined 45 metacyclic clones of seven *T. brucei *sspp. strains without finding recombinants. The use of different drug-selectable markers to distinguish two lines of the same strain also failed to reveal recombinants [4]; mixed infections were evident in two of 13 flies with infected salivary glands, but no double-drug resistant progeny were recovered.

Here we have revisited this problem using an approach that relies on the production of yellow fluorescent hybrids to indicate mating between different parental strains distinguished by red or green fluorescent proteins [7,8]. Using this robust experimental system for investigating genetic exchange in *T. brucei*, we previously demonstrated that mating took place only after trypanosomes had reached the salivary glands of the fly, and did not occur among trypanosomes in the midgut. As well as enabling hybrids to be detected by yellow fluorescence, the system makes it easy to identify which flies carry mixed populations since the two parental clones can be distinguished by red or green fluorescence. We have adapted the system to detect the occurrence of intraclonal mating by creating red and green fluorescent lines derived from a single trypanosome strain. The occurrence of yellow fluorescent trypanosomes when the red and green lines are co-transmitted through experimental tsetse flies should indicate intraclonal mating. Vice versa, transmission through tsetse flies of a yellow fluorescent trypanosome clone, which carries genes for both red and green fluorescence, would be expected to produce red and green fluorescent trypanosomes if the reporter genes segregated. Here we describe the results of both these kinds of experimental transmission.

## Methods

### Tsetse flies

Experimental tsetse flies were from the Bristol laboratory colony of *Glossina morsitans morsitans *originally from Zimbabwe. Flies were kept at 25°C and 70% relative humidity, and fed on sterile defibrinated horse blood via a silicone membrane; bloodmeals for infected flies were supplemented with 2.5% w/v bovine serum albumen (Sigma A4503) [9] and 1 mM dATP [10]. Male flies were used for experiments, being given the infective bloodmeal for their first feed 24-48 hours post-eclosion. The infective bloodmeal contained approximately equal numbers of bloodstream form (BSF) trypanosomes of each strain (approximately 8 × 10^6 ^trypanosomes ml^-1^) in sterile horse blood, or procyclics in washed red blood cells (approximately 10^7 ^trypanosomes per ml of packed cells), supplemented with 60 mM N-acetylglucosamine [11] or 10 mM L-glutathione [12] to increase infection rates.

### Trypanosomes

The trypanosome clones used were 1738 (*T. b. brucei *MOVS/KE/70/1738 [13] and J10 (*T. b. brucei *MCRO/ZM/73/J10 CLONE 1 [14]) transfected with either a gene for green fluorescent protein (*GFP*) or monomeric red fluorescent protein (*mRFP*) [15] as described previously [8]. A selectable marker gene, conferring resistance to the antibiotic hygromycin (*Hyg*) or phleomycin (*Ble*), was located downstream of the fluorescent protein gene in each plasmid construct. Constructs were targeted to the non-transcribed spacer of the ribosomal RNA locus. The clones used for mating were designated as follows according to fluorescence; the antibiotic resistance gene is given in brackets: 1738 GFP (*Hyg*), 1738 RFP (*Ble*), J10 GFP (*Ble*), J10 RFP (*Hyg*). The intraclonal crosses are referred to as 1738 RGFP and J10 RGFP. A yellow fluorescent hybrid clone, SG3 clone 7, was also transmitted through tsetse. This hybrid clone originated from a cross of J10 RFP (*Hyg*) and 1738 GFP (*Hyg*) and had a DNA content similar to that of the parental trypanosomes, 2N [8]. Procyclic forms of SG3 clone 7 were transmitted through tsetse to obtain BSF, which were subsequently retransmitted; procyclic clones were derived from both BSF populations after transformation *in vitro*.

### Dissection

Whole tsetse alimentary tracts, from the proventriculus to the hindgut, were dissected at various timepoints after feeding (21-54 days) in a drop of phosphate buffered saline (PBS) and scored for the presence of trypanosomes. A subset of these flies was used to quantify the proportion of mixed infections in the midgut by fluorescence microscopy; for the 1738 RGFP cross, trypanosomes were counted in midguts from a sample of flies dissected 21 to 32 days post infected feed, as described by [11]. Whole salivary glands from flies were dissected into a drop of PBS and viewed as wet mounts under bright field illumination to search for trypanosomes. Positive glands were then viewed by fluorescence microscopy using a DMRB microscope (Leica) equipped with a Colour Coolview camera (Photonic Science) and ImagePro Plus software (Media Cybernetics). Glands found to contain a mixed population of trypanosomes were digitally imaged before inoculation into mice. Trypanosomes from the first wave of parasitaemia were transformed to procyclics *in vitro *(see below).

### Trypanosome culture

BSF trypanosomes from mice were transformed to procyclics in Cunningham's medium [16] supplemented with 10% v/v heat-inactivated foetal calf serum, 5 μg/ml hemin and 10 μg/ml gentamycin (complete medium = CM) at 27°C and tested for drug resistance to hygromycin and/or phleomycin at 50 μg ml^-1 ^and 1 μg ml^-1 ^respectively in CM in a microtitre plate format. Plates were generally examined every 1 or 2 days for 2 weeks and individual wells passaged as necessary. Clones from transformed procyclics were obtained by limiting dilution in 96 well plates.

### Genotype analysis

Genomic DNA samples were prepared from approximately 5 × 10^7 ^washed procyclics using a spin column DNA purification kit (Qiagen). Samples for pulsed field gel (PFG) electrophoresis were prepared by lysing and deproteinising trypanosomes *in situ *in agarose blocks [17]. PFG electrophoresis, blotting and hybridization were carried out essentially as described previously [8]. Chromosomes were separated using a Biorad CHEF-DR III with a 2 phase program (Block 1: switch time 1800 s, voltage 2 V/cm, angle 106°, 15 hours; Block 2: switch time 300-900 s, voltage 3 V/cm, angle 106°, 50 hours) using 0.5 × TBE buffer and 0.9% agarose gels; gels were stained overnight by submersion in electrophoresis buffer containing ethidium bromide (2 μg/ml). Blots were hybridized with the following P^32^-labelled DNA fragments: *GFP *and *mRFP *genes from the plasmid constructs used for transfection; *β-tubulin *from cDNA plasmid clone [18]; 18S rRNA, trypanothione synthetase (*TS*; chromosome II), paraflagellar rod protein (*PFR1*, chromosome III) genes from *T. brucei *genomic DNA [19]. Microsatellite analysis was carried out using eight chromosome-specific primer sets - see Tables 3, 4, 5[20]. Primer sets for each locus were as follows: **II-PLC **5' CAACGACGTTGGAAGAGTGTGAAC, 5' CCACTGACCTTTCATTTGATCGCTTTC; **III-2 **5' GGTGGAATGGAAGATCAGTT, 5' GTTGGAATTGTTGTTGCTGT; **IV-TB4/7 **5' CCGTCACACGCCATGCACGATATG, 5' CCGTTCAGTGTGCATGTTTCAC; **VI-TB6/1 **5' CATGATGCGGAACACATGACC, 5' CTCAATAGTGCAAGTAGTCATAC; **VIII-TB8/9 **5' CCAAATATGCGATTAGTTTCC, 5' TGTTTATGTGGAAGGAAATGAA; **IX-TB9/6 **5' AAGTGTGAGGAGTTGTTGT, 5' CACCCCTTTCATCAACATCAT; **X-TB10/17 **5' CTCGCACGTTCGGATTTATGTCCG, 5' GCGACTTGTGACTTGCCTTTCTTC; **XI-53 **5' CGTGTGTCTTGTATATCTTCT, 5' TGAATAAACAAAACATGAAACGAC. Products were resolved by electrophoresis in 1 × TAE buffer through 3-5% Metaphor agarose (Cambrex) gels.

### Measurement of DNA contents

DNA contents of individual clones were measured by flow cytometry of fixed procyclics stained with propidium iodide as described [8]. Measurements were standardized by reference to the DNA content of *T. b. brucei *strain 427, arbitrarily assigned a value of 1.00; procyclics of strain 427 were included in every batch of samples run.

## Results and Discussion

### Analysis of experimental cross 1738 RGFP

For the cross 1738 RGFP, a total of 461 flies were co-infected with approximately equal numbers of BSF trypanosomes of 1738 RFP and 1738 GFP and dissected in batches 21-54 days later. The overall midgut infection rate was 43% (197/461); in a subset of these fly midguts examined by fluorescence microscopy, the majority contained a mixture of red and green fluorescent trypanosomes (78/88 = 89%), with 8% (7/88) having only green and 3% (3/88) only red fluorescent trypanosomes. Counts of trypanosomes in midguts dissected between 21 and 32 days after infection showed no significant difference in numbers of green (1.54 × 10^5 ^± 2.75 × 10^4^) and red fluorescent trypanosomes (1.61 × 10^5 ^± 1.90 × 10^4^) (*t *= 0.247, df = 18, *P *= 0.808, *n *= 19, Paired-samples *t*-test). There was thus no detectable difference in the ability of these two recombinant clones of strain 1738 to establish infection in the fly midgut.

**Figure 1:**
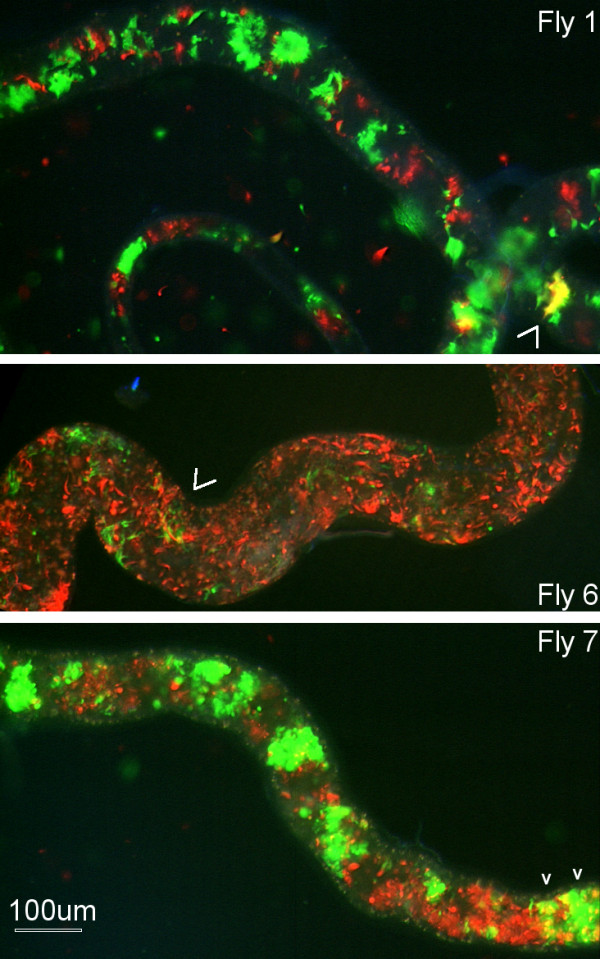
**Infected salivary glands 1738 RGFP cross**. Dissected salivary glands from three flies from 1738 RGFP cross with mixed infection of red, green and yellow trypanosomes. Arrows point to an area of gland containing yellow trypanosomes.

There were a total of 22 salivary gland infections, giving a transmission index of 11% (22/197). In seven flies, the salivary glands contained a mixed infection of red and green fluorescent trypanosomes, while the remainder had trypanosomes of only a single colour (Table 1). Small numbers of yellow trypanosomes were seen in the salivary glands of three of the seven flies with a mixed salivary gland infection (Table 2; Fig 1). This contrasts with the abundance of yellow fluorescent trypanosomes typically observed in salivary glands containing both 1738 GFP and J10 RFP in the previous interclonal cross [8].

**Table 1:** Salivary gland infections

**Cross**	**Fluorescence of trypanosomes in paired salivary glands (no. of flies)**			**Total**
		
	**Red only**	**Green only**	**Red + Green**	
1738 RGFP	2	13	7	22

J10 RGFP	9	2	2	13

**Table 2:** Composition of trypanosome population in individual flies

**Cross**	**Fly no**.	**Fluorescence of salivary gland population**^a^							**Fluoroscence of ** **transformed procyclics**	**Drug resistance of ** **population^b^**			
				
		**Gland 1**					**Gland 2**							
						
		**Red**	**Green**	**Yellow**		**Red**	**Green**	**Yellow**		**H**	**P**	**H + P**

1738 RGFP	1	++	+++	+/-		++	+++	+/-	Red	-	+	-
	
	2	+	+++	-		+	+++	-	Green	+	-	-
	
	3	+/-	+++	-		+/-	+++	-	Green	+	-	-
	
	4	+/-	+++	-		+/-	+++	-	Green	+	-	-
	
	5	+/-	+++	-		+/-	+++	-	Green	+	-	-
	
	6	++	+++	+		+++	++	+	Green, Red	+	+	-
	
	7	+/-	+++	-		+++	+++	+	Green, Red	+	+	-

J10 RGFP	1	-	-	+++		+++	-	-	Red	+	-	-
	
	2	+	+++	-		+++	+++	-	Green, Red	+	+	-

a. Only flies with mixed infections in the salivary glands are listed. +++ many, ++ some, + few, +/- very few, - no trypanosomes.

b. H = hygromycin; P = phleomycin. + growth, - no growth.

**Figure 2:**
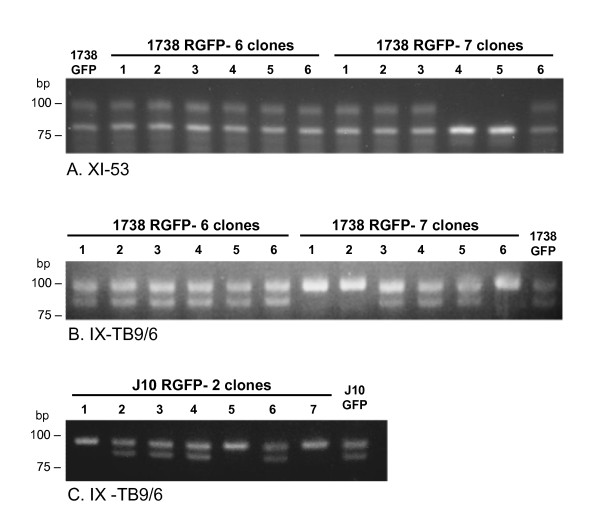
**Homozygosity demonstrated by microsatellite analysis**. PCR-amplified products for microsatellite loci indicated for the crosses 1738 RGFP (panels A and B) and J10 RGFP (panel C). Only the GFP parental clone is shown in each panel as the RFP clone was identical in each case. A. 1738 RGFP-7 clones 4 and 5 are homozygous, whereas all other clones and the parental clone, 1738 GFP, are heterozygous. B. 1738 RGFP-7 clones 1, 2 and 6 are homozygous, whereas all other clones and the parental clone, 1738 GFP, are heterozygous. C. J10 RGFP-2 clones 1, 5 and 7 are homozygous, whereas all other clones and the parental clone, J10 GFP, are heterozygous.

Metacyclics from the seven flies with mixed salivary gland infections were transformed to BSF in rodents and then into procyclic populations *in vitro *to facilitate drug selection and cloning. No yellow fluorescent trypanosomes were observed in any of the procyclic populations, with a mixture of red and green fluorescent trypanosomes evident in only two populations (Fly 6 and 7; Table 2). Growth with antibiotics showed drug resistance consistent with the observed fluorescence of the procyclic populations, but no evidence of double-drug resistant trypanosomes (Table 2). Clones were derived from populations 1, 6 and 7, all of which originated from salivary glands with red, green and yellow fluorescent trypanosomes (Table 2). Of 20 clones analysed, four from Fly 6 and 7 lacked fluorescence, while the rest were either red or green (Table 3). These clones were genotyped by microsatellite analysis of eight chromosome-specific loci (Table 3). While all clones from Fly 1 and 6 matched the 1738 genotype, five clones from Fly 7 showed homozygosity at loci where 1738 was heterozygous, indicating that they are recombinant (Table 3; Fig 2A and 2B). In each case, homozygosity was demonstrated at 3-4 independent loci, making it unlikely that these results can be explained by gene conversion.

**Table 3:** Genotypes and DNA contents of clones from 1738 RGFP cross

**Clone**	**Fluorescence**	**DNA content**^a^	**Microsatellite locus**^b^							
			
			**II-PLC**	**III-2**	**IV-TB4/7**	**VI-TB6/1**	**VIII-TB8/9**	**IX-TB9/6**	**X-TB10/17**	**XI-53**
1738 RFP	Red	0.91	cd	cd	cd	bc	bc	cd	bc	bc

1738 GFP	Green	0.91	cd	cd	cd	bc	bc	cd	bc	bc

Fly 1 clones 1-8	Red	0.90	cd	cd	cd	bc	bc	cd	bc	bc

Fly 6 clone 1	None	0.97	cd	cd	cd	bc	bc	cd	bc	bc

Fly 6 clone 2	Green	0.93	cd	cd	cd	bc	bc	cd	bc	bc

Fly 6 clone 3	Green	0.90	cd	cd	cd	bc	bc	cd	bc	bc

Fly 6 clone 4	Green	0.90	cd	cd	cd	bc	bc	cd	bc	bc

Fly 6 clone 5	Red	0.91	cd	cd	cd	bc	bc	cd	bc	bc

Fly 6 clone 6	Green	0.96	cd	cd	cd	bc	bc	cd	bc	bc

Fly 7 clone 1	Red	1.21	cd	cd	**cc**	**cc**	bc	**dd**	**bb**	bc

Fly 7 clone 2	Red^c^	0.98	cd	cd	**cc**	**cc**	bc	**dd**	**bb**	bc

Fly 7 clone 3	None	1.17	cd	cd	cd	bc	bc	cd	bc	bc

Fly 7 clone 4	None	0.96	cd	cd	**cc**	**bb**	bc	cd	bc	**bb**

Fly 7 clone 5	None	0.95	cd	cd	**cc**	**bb**	bc	cd	bc	**bb**

Fly 7 clone 6	Red	0.97	cd	cd	**cc**	**cc**	bc	**dd**	**bb**	bc

a. DNA content relative to *T. b. brucei *strain 427, arbitrarily assigned a DNA content of 1.00. For Fly 1, value given is for clone 1.

b. Primers for microsatellite loci on chromosomes II, III and XI given in [8], and on chromosomes IV, VI, VIII, IX and X from database of Dr Annette Macleod  text indicates differences from alleles of *T. b. brucei *strain 1738.

c. Two copies of *mRFP *gene

**Figure 3:**
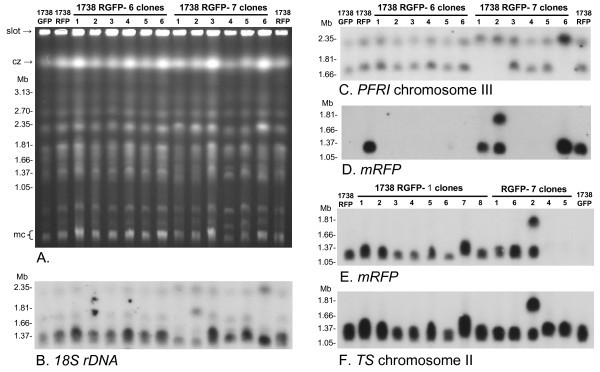
**Molecular karyotypes of clones from 1738 RGFP cross**. A. Ethidium bromide stained pulsed field gel. Size marker: chromosomal DNAs from *Hansenula wingei*. Cz = compression zone, a region of the gel where several large chromosomal bands are trapped. Mc = minichromosomes of approx. 100 kb in size. B - D. Autoradiographs of Southern blot of same gel showing results following hybridization with the probes indicated. All blots were washed to 0.1 × SSC at 65°C. E, F. Southern blot of second gel (not shown) hybridised with probes indicated. Chromosome III homologues are identical in size in Fly 7 clones 1, 2 and 6, while clone 2 has two copies of the *mRFP *gene.

This interpretation was supported by analysis of molecular karyotypes by PFG electrophoresis. Inspection of the ethidium-stained gel revealed small differences among clones from Fly 7, while clones from Fly 1 and 6 were indistinguishable from 1738 GFP or 1738 RFP; Fig 3A shows a comparison of clones from Fly 6 and 7. The variability of Fly 7 clones was confirmed by hybridization of the PFG blot with a probe for 18S rDNA (Fig 3B), which is carried on several different chromosomes (rDNA arrays on chromosomes II, III and VII [19]), and a series of chromosome-specific probes. For the *PFR1 *locus on chromosome III, where the two homologues of 1738 differ substantially in size (~1.7 and 2.2 Mb), only a single band was evident in Fly 7 clones 1, 2 and 6 (Fig 3C). As both alleles *c *and *d *were detected in these clones for microsatellite locus III-2 (Table 3), two chromosome III homologues of the same size are present; moreover, as allele *c *is normally located on the smaller of the two chromosome III homologues in strain 1738, but is now on a large homologue, chromosomal recombination has occurred. Further evidence of chromosomal recombination is the presence of two copies of the *mRFP *gene in Fly 7 clone 2, while only a single copy is present in 1738 RFP and other red fluorescent clones (Fig 3D and 3E). The *mRFP *gene was targeted to integrate into the non-transcribed spacer region of one of the rDNA arrays, and co-localization with a probe for the trypanothione synthetase (*TS*) gene showed the recombinant array to be on chromosome II (Fig 3F). While the chromosome II homologues of strain 1738 are approximately equal in size (~1.2 Mb), one homologue is much larger (~1.8 Mb) in Fly 7 clone 2 and both homologues carry the *mRFP *gene (Fig 3E and 3F).

Measurement of DNA contents by flow cytometry relative to strain 427 gave values of 0.91 for 1738 RFP and 1738 GFP, and 0.90 - 0.97 for the clones analysed from Fly 1 and 6 (Table 3). Four clones from Fly 7 also fell within or just outside this range, but clones 1 and 3 had substantially higher values of 1.17 and 1.21 respectively (Table 3). While there was evidence from microsatellite and karyotype data that Fly 7 clone 1 was recombinant, clone 3 showed no difference to the profile of 1738 except lack of fluorescence (Table 3).

Taken together, the microsatellite and karyotype data identify five clones (1, 2, 4, 5, 6) from Fly 7 as products of intraclonal mating of strain 1738. The increased DNA content of Fly 7 clone 3 also indicates that it is a recombinant. Clones 4 and 5 were identical on all criteria (Table 3), hence there are five individual genotypes from Fly 7 that result from intraclonal mating. This contrasts with the absolute fidelity of transmission of 1738 RFP and 1738 GFP as demonstrated by the recovery of unaltered clones from Fly 1 and 6.

### Analysis of experimental cross J10 RGFP

**Figure 4:**
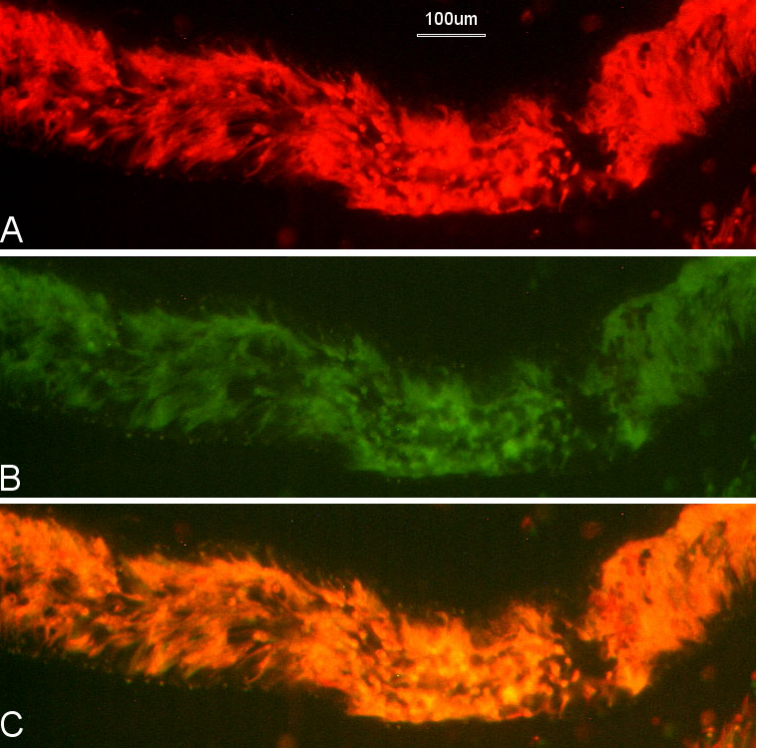
**Infected salivary gland J10 RGFP cross**. Portion of dissected salivary gland from J10 RGFP-1 containing predominantly yellow fluorescent trypanosomes. Viewed through red filter (A) or green filter (B), and merge (C).

For the cross J10 RGFP, a total of 407 flies were co-infected with approximately equal numbers of BSF trypanosomes of J10 RFP and J10 GFP and dissected in batches 33-41 days later. The overall midgut infection rate was 64% (259/407) and the majority of midguts in a subset examined by fluorescence microscopy contained a mixture of red and green fluorescent trypanosomes (51/62, 82%), with 16% (10/62) having only red and 2% (1/62) only green fluorescent trypanosomes. There were 13 salivary gland infections, giving a transmission index of 5% (13/259). For 11 of these flies, trypanosomes of single colour fluorescence were detected in both salivary glands (Table 1) and these were not analysed further. Of the two remaining flies, Fly 1 had one salivary gland packed with yellow fluorescent trypanosomes; the colour of fluorescence was verified using narrow bandwidth red and green filters (Fig 4); the other gland contained only red trypanosomes. Although we assume that yellow fluorescent metacyclics were present in the salivary gland, disappointingly no yellow fluorescent trypanosomes were observed in the procyclic population derived from the glands after mouse passage, which comprised red fluorescent trypanosomes only, and no double drug resistant trypanosomes were recovered by antibiotic selection (Table 2); BSF were not checked for fluorescence, as the fluorescent reporter genes are driven by a procyclin promotor, which is down-regulated in BSF. Fly 2 had a mixed infection of red and green fluorescent trypanosomes in both glands, but no yellow fluorescent trypanosomes were noticed, nor were double drug resistant trypanosomes recovered after antibiotic selection (Table 2).

Clones were obtained from Fly 1 and 2 procyclic populations prior to drug selection; all eight clones from Fly 1 showed red fluorescence, while those from Fly 2 showed either red or green fluorescence (Table 4). Analysis of molecular karyotypes did not reveal any differences from J10 (not shown). However, several clones showed homozygosity for the microsatellite locus on chromosome IX whereas J10 was heterozygous (Fig 2C; Table 4). A difference between J10 GFP and J10 RFP was detected for the microsatellite locus on chromosome VIII, while all clones had *ab *alleles like J10 GFP, regardless of colour of fluorescence (Table 4); we assume J10 RFP became homozygous during the selection and fly transmission involved in derivation of the lineage.

**Table 4:** Genotypes and DNA contents of clones from J10 RGFP cross

**Clone**	**Fluorescence**	**DNA content**^a^	**Microsatellite locus**^b^							
			
			**II-PLC**	**III-2**	**IV-TB4/7**	**VI-TB6/1**	**VIII-TB8/9**	**IX-TB9/6**	**X-TB10/17**	**XI-53**
J10 RFP	Red	0.96	ab	ab	ab	aa	aa^c^	ab	ab	aa

J10 GFP	Green	0.96	ab	ab	ab	aa	ab	ab	ab	aa

Fly 1 clone 1	Red	1.61	ab	ab	ab	aa	**ab**	**bb**	ab	aa

Fly 1 clone 2	Red	1.64	ab	ab	ab	aa	**ab**	**bb**	ab	aa

Fly 1 clone 3	Red	1.51	ab	ab	ab	aa	**ab**	**bb**	ab	aa

Fly 1 clone 4	Red	1.48	ab	ab	ab	aa	**ab**	**bb**	ab	aa

Fly 1 clone 5	Red	1.76	ab	ab	ab	aa	**ab**	**bb**	ab	aa

Fly 1 clone 6	Red	1.55	ab	ab	ab	aa	**ab**	**bb**	ab	aa

Fly 1 clone 7	Red	1.76	ab	ab	ab	aa	**ab**	**bb**	ab	aa

Fly 1 clone 8	Red	ND	ab	ab	ab	aa	**ab**	**bb**	ab	aa

Fly 2 clone 1	Red	1.77	ab	ab	ab	aa	**ab**	**bb**	ab	aa

Fly 2 clone 2	Green	1.05	ab	ab	ab	aa	ab	ab	ab	aa

Fly 2 clone 3	Green	0.99	ab	ab	ab	aa	ab	ab	ab	aa

Fly 2 clone 4	Green	ND	ab	ab	ab	aa	ab	ab	ab	aa

Fly 2 clone 5	Red	0.97	ab	ab	ab	aa	**ab**	**bb**	ab	aa

Fly 2 clone 6	Green	0.93	ab	ab	ab	aa	ab	ab	ab	aa

Fly 2 clone 7	Red	ND	ab	ab	ab	aa	**ab**	**bb**	ab	aa

a. DNA content relative to *T. b. brucei *strain 427, arbitrarily assigned a DNA content of 1.00. ND, not done.

b. See Table 4 for information on microsatellite loci.

c. There is an allelic difference between J10 RFP and J10 GFP, presumably generated during derivation of these separate lineages.

Measurement of DNA contents by flow cytometry relative to strain 427 gave values of 0.96 for J10 RFP and J10 GFP, but all clones from Fly 1 had greatly increased DNA contents, ranging from 1.48 to 1.76 (Table 4). Based on the haploid DNA content of J10 (0.48), these values indicate triploid cells. Fly 2 clone 1 also had a high DNA content, while the other clones measured had similar values to J10 (0.93 - 1.05; Table 4). The raised DNA contents of some clones from Fly 1 and 2, coupled with reduction to homozygosity at one microsatellite locus, suggest that these clones are products of intraclonal mating. This evidence is strengthened by the striking observation of widespread yellow fluorescence in one salivary gland of Fly 1 (Fig 4). This deserves comment, as such predominant yellow fluorescence has never been observed even in salivary glands from red/green crosses. It is possible that trypanosome fusion took place on a large scale in this gland for some reason, but this implies that large numbers of red and green fluorescent trypanosomes were already present; on the other hand, if the gland was colonized by a small population of yellow fluorescent trypanosomes, this implies that there was an actively dividing population, which does not square with the subsequent failure to recover any viable yellow trypanosomes.

### Transmission of a yellow fluorescent clone

**Figure 5:**
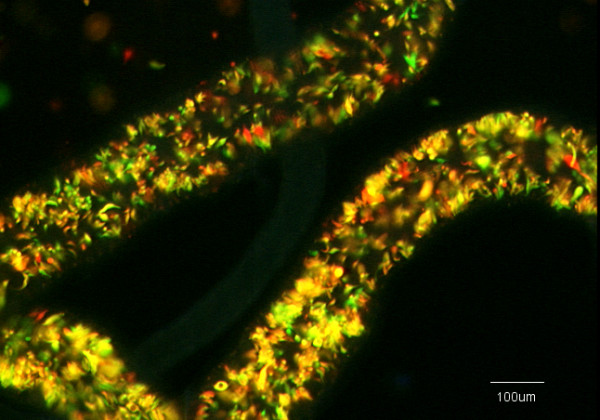
**Infected salivary glands SG3 clone 7**. Dissected salivary glands from a fly infected with the yellow fluorescent hybrid clone SG3 clone 7. Red, green and yellow fluorescent trypanosomes are present.

**Figure 6:**
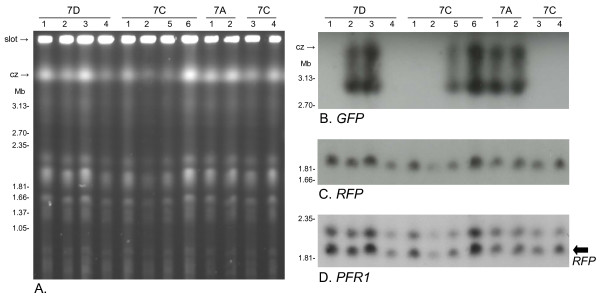
**Molecular karyotypes of clones derived after transmission of SG3 clone 7**. A. Ethidium bromide stained pulsed field gel. Size marker: chromosomal DNAs from *Hansenula wingei*. Cz = compression zone, a region of the gel where several large chromosomal bands are trapped. B - D. Autoradiographs of Southern blot of same gel showing results following hybridization with the probes indicated. All blots were washed to 0.1 × SSC at 65°C. In panel B the *GFP *gene is located on a single chromosome of approximately 3 Mb in size, but there is also hybridization to DNA trapped in the compression zone. In panel D arrow indicates chromosome III homologue that also hybridized with the *RFP *gene (panel C).

SG3 clone 7 is a hybrid clone derived from the 1738 GFP × J10 RFP cross; it expresses both *mRFP *and *GFP *genes and therefore appears yellow by fluorescence microscopy [8]. Fly transmission of this clone was carried out twice in succession, first using SG3 clone 7 procyclics in the infective bloodmeal. Three flies with infected salivary glands were obtained, but the trypanosomes were not of uniform yellow fluorescence as expected, instead showing a range of fluorescent colours including green, red and yellow (Fig 5); most surprisingly, one fly had one salivary gland of the pair containing only red fluorescent trypanosomes. The infected glands from these three flies were used to obtain BSF populations, which were transformed to procyclics *in vitro*; no green fluorescent trypanosomes were seen in this population and the red and yellow fluorescent clones obtained were analysed by microsatellite and karyotype analysis (Table 5, Transmission 1). No differences were detected in the genotypes of any of the clones except for the absence of the *GFP *gene in the red fluorescent clones (Fig 6B). The *GFP *gene was on the smaller homologue of chromosome VII in the yellow fluorescent clones; hybridization with a probe for 18S rDNA showed both chromosome VII homologues present and unchanged in size in all clones (not shown). The most likely explanation of these observations is that the *GFP *gene was removed by gene conversion, because it sits in a tandem array of rDNA repeats. However, measurement of DNA contents of two red and two yellow clones showed a range of values: while yellow clones, 7A1 and 2, both gave values of about 0.9 and are probably diploid, the two red clones had either a very high (1.58, 7D1) or low (0.78, 7D4) value (Table 5). Previously SG3 clone 7 was found to have a DNA content similar to that of its parents, 1738 GFP and J10 RFP, consistent with diploidy [8].

**Table 5:** Genotypes and DNA contents of clones from transmission of yellow fluorescent clone

**Clone**	**Fluorescence**	**DNA content**^a^	**Microsatellite locus**							
			
			**II-PLC**	**III-2**	**IV-TB4/7**	**VI-TB6/1**	**VIII-TB8/9**	**IX-TB9/6**	**X-TB10/17**	**XI-53**
SG3 clone 7	Yellow	2N^b^	bc	bd	bc	ab	ab	bc	ac	ab

Transmission 1										

7A clone 1	Yellow	0.91	bc	bd	bc	ab	ab	bc	ac	ab

7A clone 2	Yellow	0.90	bc	bd	bc	ab	ab	bc	ac	ab

7C clones 1-4	Red	ND	bc	bd	bc	ab	ab	bc	ac	ab

7C clones 5,6	Yellow	ND	bc	bd	bc	ab	ab	bc	ac	ab

7D clones 1	Red	1.58	bc	bd	bc	ab	ab	bc	ac	ab

7D clones 2,3	Yellow	ND	bc	bd	bc	ab	ab	bc	ac	ab

7D clones 4	Red	0.78	bc	bd	bc	ab	ab	bc	ac	ab

Transmission 2										

7A1-1 clones 1, 2	Yellow	ND	bc	bd	bc	ab	ab	bc	ac	ab

7A1-2 clones 1, 2	Yellow	ND	bc	bd	bc	ab	ab	bc	ac	ab

7A1-3 clone 1	Yellow	ND	bc	bd	bc	ab	ab	bc	ac	ab

7A1-3 clone 2	Red	ND	bc	bd	bc	ab	ab	bc	ac	ab

7A1-4 clone 1	Yellow	1.42	**bb**	bd	**bb**	ab	ab	bc	ac	ab

7A1-4 clone 2	Red	ND	bc	bd	bc	ab	ab	bc	**cc**	ab

7A2-1 clones 1, 3	Yellow	ND	bc	bd	bc	ab	ab	bc	ac	ab

7A2-1 clone 2	Red	ND	bc	bd	bc	ab	ab	bc	ac	ab

7A2-2 clones 1, 2	Yellow	ND	bc	bd	bc	ab	ab	bc	ac	ab

a. DNA content relative to *T. b. brucei *strain 427, arbitrarily assigned a DNA content of 1.00. ND, not done.

b. Measured previously [8].

The two yellow clones (7A1 and 7A2) were re-transmitted through flies. Of 39 flies with infected salivary glands, all except one had salivary glands with trypanosomes of red, green or yellow fluorescence as on the first transmission of the parent clone, SG3 clone 7; the exception was one fly that had only red trypanosomes in both glands. Salivary glands from six flies, representing transmission of clone 7A1 (7A1-1 to 4) and 7A2 (7A2-1 and 2) were used to obtain bloodstream form populations, which were transformed to procyclics *in vitro*. As before, no green fluorescent trypanosomes were observed in the population and only yellow or red clones were obtained for microsatellite and karyotype analysis (Table 5, Transmission 2). These clones were again indistinguishable by molecular karyotype (not shown) and most were identical by microsatellite analysis, but 7A1-4 clone 1 had become homozygous at two previously heterozygous loci and it also showed a high DNA content (1.42, Table 5). A second clone from this fly (7A1-4 clone 2) also showed homozygosity at a single microsatellite locus, but was lost prior to measurement of DNA content.

One interpretation of these results is that, like its parents 1738 and J10, SG3 clone 7 also undergoes intraclonal mating, leading to the occurrence of pure red and green fluorescent trypanosomes as well as yellow ones in the salivary glands; it is an open question whether the red and green trypanosomes are haploid intermediates or diploid progeny. As most salivary glands had this appearance, SG3 clone 7 may have a higher frequency of intraclonal mating than either parental strain, but this may also reflect the fact that these events are easy to detect in a yellow clone that carries genes for both red and green fluorescence. If so, it is puzzling that these high levels of intraclonal mating did not feed through to recovery of large numbers of recombinant progeny. Like the predominance of yellow fluorescent trypanosomes in one salivary gland for J10, the observation of salivary glands filled with red fluorescent trypanosomes in sequential transmissions of SG3 clone 7 is difficult to explain. Do these uniform populations arise by founder effects [21], or are they evidence of widespread recombination? In considering these alternatives, it is noteworthy that red and green fluorescent trypanosomes were also observed in small numbers in the midguts of infected flies.

## Conclusion

Contrary to previous reports that intraclonal mating in *Trypanosoma brucei *is only associated with out-crossing [4,5], we show here that recombinant trypanosomes are produced with relatively high frequency after fly transmission of a single strain. We have been able to demonstrate these events using mixtures of trypanosomes from a single *T. b. brucei *strain transfected with a gene for either red or green fluorescent protein. This strategy firstly enabled flies carrying a mixed infection of red and green fluorescent trypanosomes in the salivary glands to be identified, and secondly allowed us to detect any recombinants by yellow fluorescence directly in the fly [7,8]. This focused the analysis on populations likely to contain recombinants, rather than laboriously screening all populations as in previous studies. Thus, starting from 35 flies with infected salivary glands, we identified nine flies carrying a mixed infection of red and green fluorescent trypanosomes, and were able to detect putative recombinants by yellow fluorescence in four flies, of which just two finally yielded recombinant trypanosome clones. This contrasts with the high frequency of mating observed in the outcross of these two parental strains, where hybrids were easily isolated from four flies with salivary glands containing a mixed infection of red and green fluorescent trypanosomes, and putative hybrids, as judged by yellow fluorescence, were evident in a total of 17 of 22 (77%) flies with a mixed salivary gland infection [8]. Comparing the numbers of flies where yellow trypanosomes were observed in mixed salivary gland infections, at a rough estimate recombinants are half as likely to be recovered from flies carrying a single trypanosome strain (four out of nine, 44%) compared to a mixed infection (17 of 22, 77%).

What processes are occurring during intraclonal mating? The observation of yellow fluorescent trypanosomes - in abundance in the salivary glands of one fly - indicates that fusion between individual cells occurs, although we were unable to recover any yellow recombinant trypanosomes even after drug selection. This suggests that non-viable recombinants may sometimes be formed, echoed by the observation that salivary glands from flies infected with a yellow fluorescent clone often appeared to contain a mixture of red, green and yellow fluorescent trypanosomes but green fluorescent trypanosomes were not recovered. One possibility is that a proportion of trypanosomes undergo meiosis, but the resultant intermediates then fail to produce viable diploids. Perhaps the lack of cells of a different mating type in a single strain transmission aborts the normal course of events and the intermediates simply die *in situ*. As mentioned earlier, there is currently no evidence that mating types occur in *T. brucei*, but the contrasting numbers of recombinant/non-recombinant clones recovered from single versus two strain transmissions continues to support the idea that trypanosomes can recognise self and non-self.

After fly transmission, it was found that several clones analysed had a raised, or in one case, lowered DNA content relative to the original clone prior to fly transmission. Most of these clones also had a recombinant genotype according to microsatellite analysis, but, in three cases, no change other than in DNA content was detected, although more extensive genotyping might reveal evidence of recombination. Some of the raised DNA contents observed were consistent with triploidy or tetraploidy, but the molecular karyotype analysis revealed no evidence of extra chromosomes. This echoes the finding of hybrids with raised DNA contents in the previous outcross, where it was suggested that the putative 4N hybrids might have been formed by fusion of unreduced gametes [8].

Although the overall picture is far from clear, one thing is certain: that it is no longer possible to assume that strains are fly-transmitted unaltered. This has implications for both laboratory and field infections, as infected glands will potentially contain a heterogeneous population of trypanosomes.

## Competing interests

The authors declare that they have no competing interests.

## Authors' contributions

WG, LP and MB designed the study. All authors contributed to the experimental work. WG and LP drafted the manuscript. All authors read and approved the final manuscript.
